# Analysis of the Uptake of Hypericin by *Candida albicans* Yeast Cells Using Fluorescence Methods and Comparison of the Dynamics of This Process over Time

**DOI:** 10.3390/pharmaceutics18020189

**Published:** 2026-01-31

**Authors:** Radosław Turski, Jakub Fiegler-Rudol, Hanna Hüpsch-Marzec, Dariusz Skaba, Rafał Wiench

**Affiliations:** Department of Periodontal and Oral Mucosa Diseases, Faculty of Medical Sciences in Zabrze, Medical University of Silesia, 40-055 Katowice, Poland; s88998@365.sum.edu.pl (J.F.-R.); hanna.hupsch-marzec@sum.edu.pl (H.H.-M.); rwiench@sum.edu.pl (R.W.)

**Keywords:** *Candida albicans*, antimicrobial photodynamic therapy, fluorescence microscopy, hypericin, image analysis, kinetics, LED illumination, photosensitizer uptake

## Abstract

**Background**: Hypericin is a natural photosensitizer with promising antifungal potential, but its uptake kinetics in *Candida* (*C*.) *albicans* are not well defined. **Objective**: To characterize the time-dependent uptake of hypericin by *C. albicans* in vitro using fluorescence microscopy and quantitative image analysis. **Methods**: *C. albicans* ATCC 90028 was standardized to 0.5 McFarland and incubated with hypericin dissolved in DMSO. Samples were illuminated with an LED system tuned near 550 nm and imaged using a CCD fluorescence microscope with emission recorded above ≈600 nm. Images were analyzed in ImageJ, using a control-based threshold and percentage area (the percentage of pixels above the threshold in the whole field) as a fluorescence measure. Time points were 1, 3, 5, 7, 10, 15, 20, 25, 30, 35, 40, and 45 min, plus a separate dark-only series at 35–45 min. Data from three experiments were evaluated by ANOVA. **Results**: Fluorescence increased rapidly and showed a nonlinear, biphasic profile under light, with local maxima around 5–7 and 15–30 min. Dark-only samples at 35–45 min had a lower %Area and lacked a clear biphasic pattern. **Conclusions**: Hypericin uptake by *C. albicans* is dynamic, nonlinear, and light-dependent. These kinetics should be considered when designing hypericin-based antifungal photodynamic therapy protocols.

## 1. Introduction

Fluorescence occurs when a molecule absorbs light, briefly elevates an electron to a higher energy state, and then emits a lower energy photon as it returns to the ground state, producing a Stokes shift [1]. Ring-structured molecules, such as hypericin, contain delocalized pi electrons that absorb light efficiently and readily generate fluorescence [1,2,3]. These pi-rich conjugated systems also support stable excited states that enable photodynamic production of reactive oxygen species important in therapy [2,3]. Longer conjugation lengths increase emission wavelength and enhance fluorescence efficiency [2]. Fluorescence is widely used in biology and medicine, as it enables the observation of molecular and cellular processes in real time. In clinical practice, it is applied in fluorescence microscopy, cancer diagnostics, intraoperative imaging, and dermatology. In dentistry, fluorescence is used mainly for diagnostic purposes, while photodynamic therapy (PDT) serves therapeutic functions. Both approaches are gaining importance. Fluorescence allows early detection of dental caries and the monitoring of demineralization and remineralization processes through methods such as QLF and DIAGNOdent [3,4,5]. Red fluorescence emitted by bacterial porphyrins is also used to visualize dental plaque and biofilm activity [6]. Fluorescent staining techniques can support the diagnosis of oral fungal infections, including those caused by *Candida* species, reducing the time required compared to standard laboratory procedures [7,8,9].

For fungal infections such as denture stomatitis, photodynamic therapy has proven effective and, when combined with conventional treatment, even more efficient than pharmacotherapy alone [10]. In the management of chronic periodontitis, PDT used as an adjunct to scaling and root planning can improve clinical parameters, including bleeding on probing (BOP), pocket depth (PD), and clinical attachment level (CAL) [11]. A broad review of the literature confirms that applications of PDT in dentistry are rapidly expanding and now include endodontics, as well as the treatment of oral precancerous lesions and oral cancers [12,13].

Fluorescence-based methods are widely used in biomedical research to investigate molecular localization and time-dependent cellular processes [1,2,3]. In the context of photodynamic therapy (PDT), fluorescence additionally enables noninvasive monitoring of photosensitizer distribution and accumulation, which are key determinants of therapeutic effectiveness [2,4].

Antimicrobial photodynamic therapy (aPDT) relies on the activation of a photosensitizer by light to generate reactive oxygen species that damage microbial cells [14,15]. While lasers have traditionally been used as light sources, light-emitting diode (LED) systems are increasingly applied due to their stability, accessibility, and suitability for wavelength-specific activation [16]. Importantly, light exposure itself may influence photosensitizer behavior, including aggregation state, fluorescence efficiency, and cellular uptake, highlighting the need for time-resolved assessment under illumination conditions relevant to aPDT. Natural compounds have gained growing attention in pharmaceutical and biomedical applications due to their favorable biocompatibility, structural diversity, and long-standing history of human exposure [13]. Among them, hypericin, a naturally occurring anthraquinone pigment isolated from *Hypericum perforatum*, exhibits strong absorption in the visible range, intense fluorescence emission, and high photodynamic activity [1,13]. These properties have led to extensive investigation of hypericin in oncology and dermatology, as well as increasing interest in its antimicrobial potential [14,17,18,19].

**Figure 1:**
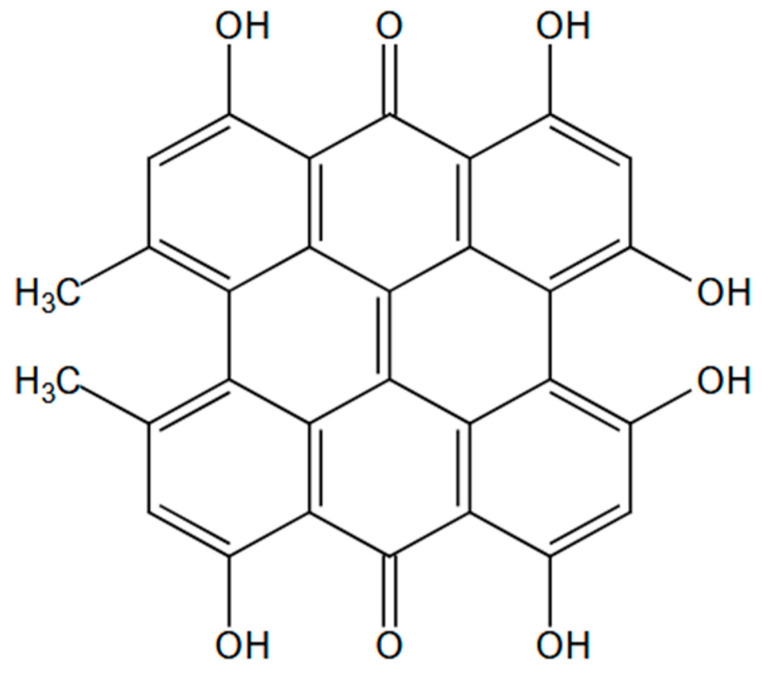
The structure of Hypericin.

Hypericin (C_30_H_16_O_8_, Figure 1) is a natural anthraquinone pigment isolated from *Hypericum perforatum*. Its structure contains a polyhydroxylated system of aromatic rings with conjugated double bonds and numerous π electrons that give rise to its photoactive and fluorescent properties [13]. The extensive cloud of delocalized π electrons enables strong absorption of visible light, which accounts for its intense red color, and makes hypericin a highly effective photosensitizer.

Despite substantial evidence supporting the photodynamic activity of hypericin, limited information is available on its uptake kinetics in fungal cells. In particular, the time-dependent accumulation of hypericin in *Candida albicans* under LED-based illumination conditions has not been systematically characterized. Understanding these kinetics is essential, as photosensitizer uptake and intracellular availability directly influence photodynamic outcomes and determine optimal incubation times in aPDT protocols [15].

*Candida albicans* was selected in this study as a model yeast organism for investigating photosensitizer accumulation. Its well-characterized cell wall structure, known transport and efflux mechanisms, and widespread use in photodynamic research make it suitable for controlled kinetic and methodological studies [7,8,9]. Importantly, the present work does not aim to directly extrapolate to clinical efficacy but rather to use *C. albicans* as an experimental system for studying fundamental aspects of hypericin accumulation and fluorescence behavior.

The aim of this study was therefore to investigate the time-resolved uptake of hypericin by *Candida albicans* using fluorescence microscopy and quantitative image analysis under LED illumination. Hypericin was examined both as a photosensitizer of pharmaceutical relevance and as an intrinsic fluorescent probe enabling noninvasive monitoring of accumulation dynamics over time. By combining controlled incubation, defined illumination, and threshold-based fluorescence analysis, this work addresses a methodological gap in current photodynamic research.

## 2. Materials and Methods

### 2.1. Organisms and Growth Conditions

The study was conducted using a reference strain of *Candida* obtained from the American Type Culture Collection (ATCC, Manassas, VA, USA): *Candida albicans* ATCC 90028. The strain was cultured on Sabouraud dextrose agar (SDA) supplemented with 4 percent glucose (BTL, Lodz, Poland) and incubated under atmospheric conditions at 37 °C. After 24 h of incubation, a colony sample was removed from the agar surface and suspended in sterile physiological saline (0.9 percent NaCl). The density of the working suspension was measured using a Densimat densitometer (bioMérieux, Marcy l’Etoile, France) and standardized to McFarland 0.5. In contrast to bacterial suspensions, McFarland standards correspond to lower cell counts in yeasts; McFarland 0.5 is typically equivalent to approximately 1 × 10^6^–5 × 10^6^ *Candida* cells/mL, as reported in CLSI M44-A guidelines and the supporting literature [20].

### 2.2. Hypericin Solution

Hypericin powder (Sigma-Aldrich, St. Louis, MO, USA) was dissolved in DMSO (dimethyl sulfoxide) (Biomus, Lublin, Poland) at a concentration of 5 mg per 5 mL. The solution was stored in darkness in a glass container at room temperature. Initial attempts to use ethanol as a solvent (based on pilot tests) indicated its limited solubility and cytotoxicity toward yeast cells. Similar findings have been reported in the literature, showing that ethanol at higher concentrations (2.5 percent v/v or above) is cytotoxic to cell lines and that ethanol extracts of *Hypericum perforatum* are toxic irrespective of light exposure [21,22,23]. For this reason, DMSO was selected as the solvent. The stock solution corresponded to 1 mg/mL hypericin in DMSO.

### 2.3. Cell Preparation and Hypericin Incubation

To prepare samples, 50 µL of the yeast suspension was pipetted using a ChemLand pipette (Przedsiębiorstwo Techniczno-Handlowe Chemland, Stargard Szczeciński, Poland) with disposable tips onto a microscope slide, followed by the addition of 5 µL of the hypericin solution in DMSO. A coverslip was placed on top, and excess liquid was removed using absorbent lignin paper. For the control images, no hypericin solution was added to the yeast suspension. The preparations were kept in complete darkness except during image acquisition. After mixing 50 µL of yeast suspension with 5 µL of hypericin stock, the final hypericin concentration in the preparation was 0.091 mg/mL (≈0.18 mM), and the final DMSO concentration was 9.1% (*v*/*v*).

### 2.4. Light Source

Illumination was provided by a set of seven green 5 mm LED diodes with an approximate wavelength of 570 nm according to the manufacturer (Botland-OEM, Bralin, Poland). Measurements performed with a Hopocolor HPCS-310P spectrophotometer (Hangzhou Hopoo Light and Color Technology Co., Ltd., Hangzhou, China) showed a maximum irradiance (µW/cm^2^) at 518 nm, with a spectral range from 460 nm to 600 nm. To obtain a spectrum closer to 550 nm, an additional green filter (550 nm ± 5 nm) from Neemoo (Shenzhen Kaitao Optical Technology Co., Shenzhen, China) was used. The maximum irradiance shifted to 532 nm, and at approximately 550 nm, the irradiance reached 0.372 µW/cm^2^/nm.

### 2.5. Microscopic Registration of Hypericin Absorption by Candida albicans Cells

Hypericin is a photosensitizer that is difficult to evaluate optically, and direct observation of its uptake by yeast cells under standard light microscopy is practically impossible. For this reason, fluorescence emission was induced using a narrow light band centered around 550 nm. Illumination was provided by a set of LED diodes with a declared emission peak at 570 nm, combined with a narrow band 550 nm filter.

Fluorescence imaging was performed using a microscope equipped with a CCD camera (ASI ZWO294MC, Suzhou ZWO Co., Ltd., Suzhou, China) with a 2x Focal Extender (Explore Scientific LLC, Springdale, AR, USA) and licensed SharpCap software(version 4.1) (AstroSharp, Alamo, Nottinghamshire, UK). The software was configured to record hypericin emission above approximately 600 nm. Images were saved in PNG format, which allowed later analysis of RGB channels [24]. Constant exposure, gain, and white balance settings were used to ensure comparability across all images.

### 2.6. Image Analysis

Image analysis was performed in ImageJ version 1.54d (National Institutes of Health, Bethesda, MD, USA) according to the measurement procedure established in this study [25]. First, the RGB channels were split (Image → Color → Split Channels), and the red channel was selected for further evaluation. Based on the control image, the fluorescence threshold was determined (Image → Adjust → Threshold). The lower threshold value was recorded (with the upper threshold set to the maximum value of 255) and used consistently across all image series.

For the time series (1, 3, 5, 7, 10, 15, 20, 25, 30, 35, 40, and 45 min, following a measurement scheme similar to protocols used for other photosensitizers against *Candida* [26], as well as 35, 40, and 45 min for the dark series), the Analyze Particles function was applied (Analyze → Analyze Particles) with Size set to 0–Infinity and with Display Results and Summarize enabled. The Total Area column provided the number of pixels above the established threshold, and the %Area column indicated the percentage of fluorescence within the entire image [27]. Values near 0 percent in the control corresponded to the background. Increases in %Area at later time points indicated greater uptake of hypericin by the cells.

### 2.7. Statistical Analysis

Statistical analysis was performed in LibreOffice Calc (The Document Foundation, Berlin, Germany; version 25.8.1.1). Statistical analysis was performed using one-way analysis of variance (ANOVA) to evaluate the effect of incubation time on fluorescence area (%Area). Multiple pairwise comparisons between time points were conducted using Tukey’s honestly significant difference (HSD) post hoc test to control the family-wise error rate. A *p*-value ≤ 0.05 was considered statistically significant.

## 3. Results

**Figure 2:**
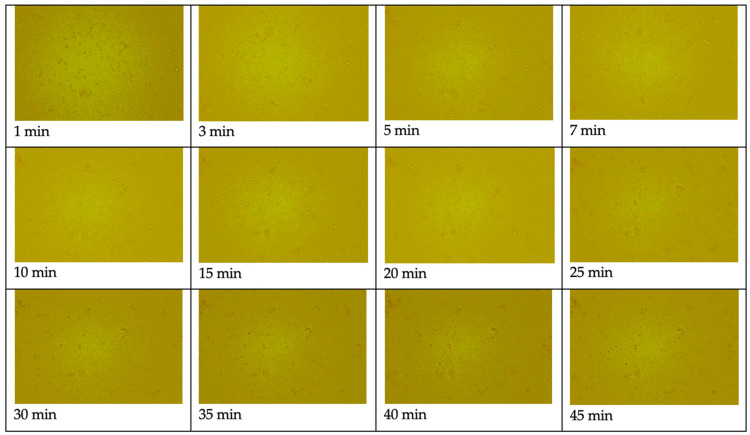
Time series of fluorescence micrographs illustrating hypericin uptake in yeast cells at 1, 3, 5, 7, 10, 15, 20, 25, 30, 35, 40, and 45 min. Images correspond to the expanded second stage of the experiment and were used for quantitative ImageJ analysis of fluorescence area (%Area) following a fixed threshold based on the control sample (Magnification ×600).

**Figure 3:**
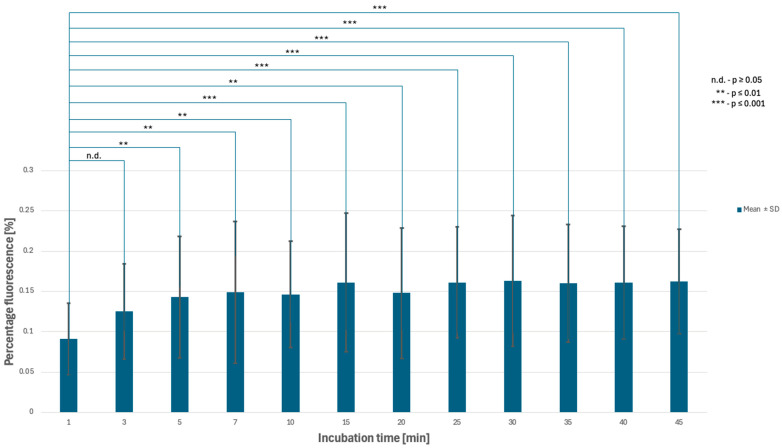
Kinetics of hypericin fluorescence in *Candida albicans* under in vitro conditions.

In the first phase of the study, a control image (control sample) was acquired to establish the threshold of possible fluorescence, reflecting autofluorescence associated with endogenous fluorophores in yeast cells without hypericin. The fluorescence threshold was set at 0.076 (%Area), which served as a reference for all subsequent analyses. In the second phase, images were taken at 1, 3, 5, 7, 10, 15, 30, and 45 min. Visual inspection suggested non-monotonic changes in fluorescence over time; however, variability between independent trials limited consistent statistical confirmation of a biphasic kinetic profile. Based on these observations, the second stage was repeated with additional images at 20, 25, 35, and 40 min. Below are images illustrating the expanded second stage, showing hypericin-stained yeast cells at 1, 3, 5, 7, 10, 15, 20, 25, 30, 35, 40, and 45 min (Table 1, Figure 2 and Figure 3).

**Figure 4:**
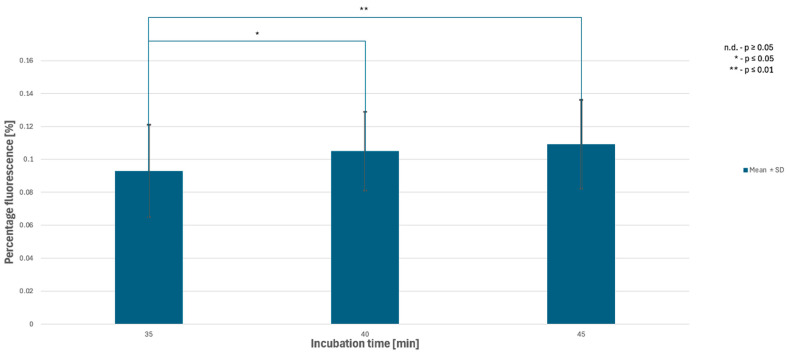
Kinetics of hypericin fluorescence in *Candida albicans* under dark conditions.

One-way ANOVA indicated that incubation time significantly affected fluorescence area in illuminated samples (overall time effect, *p* < 0.05). Tukey’s HSD post hoc analysis demonstrated that later incubation intervals (15–45 min) exhibited significantly higher %Area values compared with early time points (1–3 min) (adjusted *p* < 0.05). The results demonstrated a nonlinear pattern of cellular saturation, with discrepancies appearing between 30 and 45 min. Two of the trials showed decreases in hypericin fluorescence at these time points. To confirm or exclude a possible light-related effect, three independent series were performed only at 35, 40, and 45 min, without prior exposure to imaging at earlier time points. These were the dark trials. The Kinetics of hypericin fluorescence are shown in Figure 3 and Figure 4.

**Figure 5:**
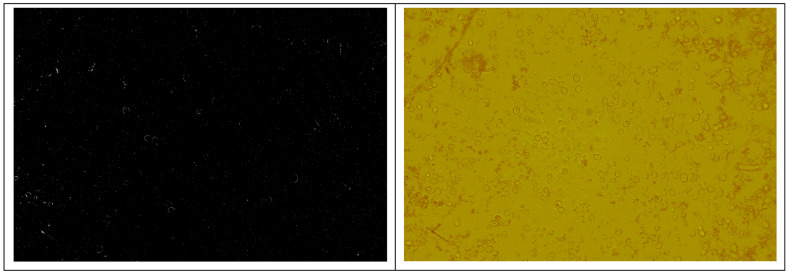
Representative dark-series micrographs illustrating minimal and subtle fluorescence signals in yeast cells in the absence of light activation. Owing to the low contrast between background and cellular fluorescence, visual differentiation was limited, and quantitative evaluation required threshold-based image analysis using specialized software (Magnification ×600).

All three dark series, like the earlier ones, produced images that were difficult to analyze without specialized software because the fluorescence differences were subtle (Figure 5, Table 2).

Although a non-monotonic temporal fluorescence profile was visually observed, substantial inter-experimental variability was present, particularly with Trial 3 exhibiting consistently higher absolute %Area values than Trials 1–2. Data were not normalized prior to analysis, as the objective was to preserve absolute fluorescence signal differences between independent experiments. Therefore, the reported kinetics should be interpreted as fluorescence accumulation dynamics rather than a definitively confirmed biphasic uptake model. One-way ANOVA demonstrated a significant overall effect of incubation time on fluorescence area (*p* < 0.05); however, due to variability and the limited number of replicates (n = 3), conservative post hoc comparisons between individual time points did not consistently support discrete biphasic peaks. Further studies with increased replication and complementary quantitative uptake assays are required to confirm nonlinear kinetic phases.

## 4. Discussion

In the light-exposed series, a two-phase pattern was observed: a rapid increase in fluorescence during the first minutes, followed by a decrease and a secondary local maximum at around 15 and 25–30 min, then a decline. In the dark-only series (35–45 min), the pattern was more stable, without pronounced saturation and with significantly lower fluorescence at all three time points compared with the illuminated series [28,29]. A transient decrease in fluorescence after initial dye uptake has been reported for toluidine blue and methylene blue, where it was attributed to efflux activity and changes in membrane condition [29].

Photosensitizers used in antimicrobial photodynamic therapy differ in uptake kinetics and optimal incubation times due to variations in molecular size, charge, solubility, aggregation behavior, and interactions with fungal cell membranes and efflux systems. Hypericin, a naturally derived anthraquinone from Hypericum perforatum, exhibits time-dependent accumulation that is strongly influenced by its aggregation state and microenvironment, resulting in non-linear uptake profiles [13,19]. Synthetic cationic dyes such as toluidine blue O and methylene blue typically show rapid surface binding and fast initial uptake, allowing effective aPDT after short incubation periods; however, their intracellular retention may be limited by active efflux and photobleaching, leading to early signal plateaus or declines [26,27,28,29,30]. In contrast, natural photosensitizers such as curcumin and riboflavin display slower and formulation-dependent uptake kinetics, with incubation time playing a critical role in achieving sufficient intracellular availability before illumination [31,32]. Berberine represents a distinct model in which uptake kinetics are additionally shaped by interference with efflux pump activity, resulting in enhanced intracellular retention over time [33,34,35]. Compared with these agents, hypericin demonstrates intermediate uptake kinetics, with clinically realistic incubation times ranging from several to several dozen minutes. Its non-linear accumulation pattern likely reflects the combined effects of membrane partitioning, aggregation–monomer transitions, light exposure, and efflux activity. These characteristics underscore the importance of incubation-time optimization when designing hypericin-mediated aPDT protocols and justify direct kinetic assessment rather than reliance on fixed incubation intervals derived from other photosensitizers [32,33,34,35,36,37].

Three main methodological categories appear in the literature. Imaging-based approaches include fluorescence microscopy (including confocal), spectrofluorometry, and whole-cell absorption measurements, which directly assess photosensitizer influx and retention over time, as shown in studies on TBO and hypericin localization [38]. Biological assays evaluate aPDT effectiveness through CFU counts, viability tests (XTT/MTT), biofilm biomass and viability, and ROS measurements, with incubation time treated as a protocol variable and outcomes defined by microbial reduction [39]. Mixed and optimization-oriented methods combine photosensitizers (for example, MB plus KI), use design of experiments strategies, incorporate spectral and photophysical analysis (photobleaching, monomer-to-aggregate transitions), and apply carrier modifications, such as PVP, to improve hypericin stability and performance [30,38].

Differences between dark and light conditions extend beyond phototoxicity. Light can modulate the aggregation state of hypericin and its microlocalization (membrane versus cytoplasm), which influences fluorescence efficiency. Carriers and polymers such as PVP increase the emission and photophysical stability of hypericin, suggesting that biological matrices containing proteins and lipids may induce similar effects [38]. Efflux pump activity and membrane condition can also transiently reduce the signal and create hump-shaped kinetic profiles.

Although lasers dominated classical PDT, LED systems are now recognized as fully adequate therapeutic light sources due to their low cost, simplicity, and the ability to select narrow spectral bands. For hypericin, effective activation is achieved with wavelengths in the yellow-to-orange range (about 550–600 nm), which makes LEDs near 590 nm suitable. Studies report successful hypericin activation and biofilm reduction using LEDs in this spectral window with incubation times of 20 to 30 min [36,37,40].

This study has several limitations. A single-organism model was used, as only *C. albicans* was examined without comparison to non-albicans *Candida* species or to multispecies biofilms [28]. Fluorescence was used as a proxy endpoint for uptake rather than a direct measure of antimicrobial efficacy, since CFU, viability, and ROS production were not assessed; the signal therefore reflects dye accumulation rather than microbial elimination [39]. Environmental factors such as carriers (for example, PVP), salinity, pH, or proteins were not modulated, although they can markedly influence hypericin aggregation and fluorescence brightness [38]. Efflux modulation was not investigated, as inhibitors of efflux pumps (such as verapamil or berberine-based modulators) were not included, which would be important to directly test the efflux-related hump hypothesis described for other dyes and analogues [41]. The relatively high final DMSO concentration represents an additional limitation, emphasizing the importance of solvent controls and future formulation improvements.

After optimization of incubation time and light parameters, hypericin may expand the panel of photosensitizers used to treat *Candida* infections in denture stomatitis and oral biofilms. There are successful examples of aPDT with other photosensitizers (MB, TBO, CUR), which indicates a realistic path toward implementation [28,39]. Hypericin is considered one of the most promising natural photosensitizers due to its efficacy against oral microorganisms and its biophysical properties demonstrated in in vitro models. The accumulation data presented in this study may serve as a starting point for designing protocols for premalignant and malignant lesions in the oral cavity, as well as for studies on immunomodulation [32].

Two-track validation of the effect: combining fluorescence with viability tests (CFU, XTT/MTT) and ROS determination (DCFH DA) would allow correlation of the sequence “accumulation → ROS → reduction in cell numbers” [39]. Control of the aggregation state: assessing the spectrum and quantum yield of hypericin under the experimental conditions and testing carriers (PVP) and proteins (for example, albumin) may smooth out the observed “humps” and strengthen the signal [38]. The inclusion of efflux pump inhibitors (for example, verapamil and comparison with berberine as a resistance modulator) would make it possible to test the “efflux hump” hypothesis [41]. Resting-pulsed schemes (for example, a short pulse of about 7 min, breaks, followed by exposures at 15 and 30 min) and dose-time relationships (J/cm^2^ versus minutes) are suggested by optimization-oriented aPDT studies [30].

Future work should broaden the biological scope by including non-*albicans Candida* species and clinical isolates obtained from dental practice, transition from planktonic cells to 24–48 h biofilms, including mixed species communities [42], and refine protocol parameters by mapping the relationship between incubation time, hypericin concentration, and light exposure (LED 590 nm in continuous and pulsed modes) with CFU and ROS as key outcomes [43]. After in vitro optimization, the next step will involve ex vivo and in vivo studies, followed by a pilot randomized controlled trial in denture wearers with oral yeast lesions, comparing hypericin-based protocols with nystatin or azoles using both clinical and microbiological endpoints.

## 5. Conclusions

Hypericin accumulated in *Candida albicans* under in vitro conditions in a manner that depended on incubation time and light exposure, with a dynamic and nonlinear uptake pattern marked by an early rise in fluorescence, a subsequent decrease, and a secondary increase in illuminated samples, while dark controls showed consistently lower signals and no biphasic behavior. These findings indicate that light near 590 nm not only enables detection of hypericin fluorescence but also influences its cellular accumulation. The divergence between the illuminated and dark series suggests that light facilitates dye influx or alters its fluorescent forms, potentially through mild photodynamic effects on the membrane, while the initial rise in signal likely reflects binding to the cell wall or membrane before internalization and the conversion of aggregates into more fluorescent monomers. Transient fluorescence humps may stem from temporary activation or saturation of efflux pumps, such as Cdr1, Cdr2, or Mdr1, and short imaging exposures may further modify membrane permeability. Methodologically, light parameters must be treated as experimental variables, dark controls are essential, and photobleaching or photoactivation should be reported. Clinically, hypericin shows promise as an alternative or adjunct photosensitizer for treating oral fungal infections and defining optimal incubation and illumination settings could support future in vitro protocols and early-stage clinical studies. The results justify expanded research on additional *Candida* species and clinical isolates, biofilm models, full microbiological validation using CFU and viability assays, and comparisons with other natural photosensitizers, such as curcumin, riboflavin, and berberine.

## Figures and Tables

**Table 1:** Dynamics of hypericin fluorescence expressed as %Area above threshold.

Time	1 min	3 min	5 min	7 min	10 min	15 min	20 min	25 min	30 min	35 min	40 min	45 min
Trial 1	0.056	0.084	0.091	0.090	0.092	0.098	0.097	0.112	0.111	0.111	0.117	0.106
Trial 2	0.078	0.098	0.109	0.107	0.127	0.126	0.106	0.131	0.122	0.125	0.124	0.146
Trial 3	0.140	0.192	0.229	0.251	0.220	0.259	0.242	0.239	0.256	0.243	0.242	0.233
Mean	0.091	0.125	0.143	0.149	0.146	0.161	0.148	0.161	0.163	0.160	0.161	0.162
SD	0.044	0.059	0.075	0.088	0.066	0.086	0.081	0.069	0.081	0.073	0.070	0.065

**Table 2:** Dynamics of hypericin fluorescence in dark conditions (%Area above threshold).

Time	35 min	40 min	45 min
Trial 1 (Dark)	0.061	0.077	0.078
Trial 2 (Dark)	0.110	0.119	0.122
Trial 3 (Dark)	0.108	0.118	0.127
Mean (Dark)	0.093	0.105	0.109
SD (Dark)	0.028	0.024	0.027

## Data Availability

The original contributions presented in this study are included in the article. Further inquiries can be directed to the corresponding authors.
